# Association between food insecurity and major depressive episodes amid Covid-19 pandemic: results of four consecutive epidemiological surveys from southern Brazil

**DOI:** 10.1017/S1368980021004626

**Published:** 2022-04

**Authors:** Leonardo Pozza Santos, Antônio Augusto Schäfer, Fernanda Oliveira Meller, Inacio Crochemore-Silva, Bruno Pereira Nunes, Jenifer Harter, Débora da Cruz Payão Pellegrini, Christian Loret de Mola

**Affiliations:** 1 Department of Nutrition, Federal University of Pelotas, Pelotas 96010-610, Brazil; 2 Postgraduate Program in Public Health, University of Southern Santa Catarina, Criciúma, Brazil; 3 Postgraduate Program in Physical Activity, Federal University of Pelotas, Pelotas, Brazil; 4 Postgraduate Program in Nursing, Federal University of Pelotas, Pelotas, Brazil; 5 College of Nursing, Federal University of Pampa, Uruguaiana, Brazil; 6 Postgraduate Program in Animal Science, Federal University of Pampa, Uruguaiana, Brazil; 7 Postgraduate Program in Public Health, Federal University of Rio Grande, Rio Grande, Brazil

**Keywords:** Food security, Mental health, Depression, Pandemics, Covid-19

## Abstract

**Objective:**

To assess the association between household food insecurity (FI) and major depressive episodes (MDE) amid Covid-19 pandemic in Brazil.

**Design:**

Cross-sectional study carried out with data from four consecutive population-based studies.

**Setting:**

The study was conducted between May and June 2020, in *Bagé*, a Brazilian southern city. Household FI was measured using the short-form version of the Brazilian Food Insecurity Scale. Utilising the Patient Health Questionnaire-9, we used two different approaches to define MDE: the cut-off point of ≥ 9 and the diagnostic criteria proposed by the Diagnostic and Statistical Manual of Mental Disorders (DSM-IV-TR). Association between FI and MDE was analysed using crude and adjusted Poisson regression models.

**Participants:**

1550 adults (≥ 20 years old).

**Results:**

The prevalence of household FI was 29·4 % (95 % CI 25·0, 34·4). MDE prevalence varied from 4·4 % (95 % CI 3·1, 6·0), when we used the DSM-IV-TR criteria to define this condition, to 9·6 % (95 % CI 7·3, 12·5) of the sample, when we used the cut-off point of ≥ 9 as definition. The prevalence of MDE was more than two times higher in those individuals living with FI, independent of the criteria adopted to define the outcome. Adjustment for potential confounders did not change the association’s magnitude.

**Conclusions:**

Household FI has been positively associated with MDE amid Covid-19 pandemic, independent of socio-demographic characteristics of participants. Actions are needed to warrant basic living conditions to avoid FI and hunger and its consequences for the Brazilian population, especially those consequences linked to mental health disorders.

Depressive disorders are one of the most important public health issues nowadays^(1,2)^. They are associated with individual and collective distress, increasing the risk of adverse outcomes and early death^(3–5)^. According to the WHO, over 300 million people experienced depression worldwide, which is equivalent to 4 % of the world’s total population^(6)^. In addition, the number of all-age years lived with disability attributable to depressive disorders increased by around 14 % from 2007 to 2017^(7)^. In Brazil, the scenario is not different; nationally representative data from different surveys estimated a prevalence of major depressive episodes (MDE) by around 4 %^(8,9)^, with significant differences among the country’s regions.

The Covid-19 pandemic, which started in March 2020, can sharply increase mental health disorders^(10,11)^, mainly in settings that opted to mitigate instead eliminate the virus. Non-pharmacological strategies to mitigate virus transmission appear to be worse for health and economy when compared with strategies aiming elimination of the severe acute respiratory syndrome coronavirus 2 (SARS-CoV-2)^(12)^. Besides those non-pharmacological strategies to mitigate virus transmission, financial problems as well as changes in household routine and individual’s lifestyle may exacerbate the incidence of mental health disorders like stress and depression^(13)^. Investigators have demonstrated increases in the prevalence of mental health disorders amid Covid-19 pandemic in different countries^(14–16)^.

Along with mental health disorders, food insecurity (FI) – which is present when people have no access to sufficient safe and nutritious food to meet their dietary needs and food preferences for an active and healthy life, according to the FAO^(17)^ – is another consequence of the Covid-19 pandemic. FAO has warned that the number of people experiencing FI might double from 2019 to 2020 due to the effects of the Covid-19 pandemic on economic and labour market^(18)^. Mental health disorders are among the main consequences of FI on people’s health as the association between household FI status and mental illness is well reported in the literature. A study that analysed the association between FI and mental disorders in 149 countries showed that individual-level FI status was associated with poorer mental health^(19)^. Several other studies have already linked FI status to the risk of mental health disorders in diverse contexts and populations^(20–22)^.

Increase in household FI has been observed in Brazil in the last years as a consequence of the political-economic crisis experienced by the country since 2015^(23,24)^, after a long period of upsurging in this situation with improvements in Brazilian dietary intake patterns^(25,26)^. The pandemic frame in Brazil, which is one of the worst countries in the outbreak coping^(27)^, may further increase FI situation all over the country, with impacts on population mental’s health.

However, if FI is linked to higher risk of mental health problems, the opposite seems to be also true. A narrative review of longitudinal studies has evidenced that the relationship between household FI status and emotional health may operate in both ways^(28)^. In other words, poor mental health could also increase the risk of FI status, which makes studying this relationship even more complex. Increase in FI caused by Covid-19 outbreak may aggravate mental health situation in the pandemic context and vice versa, mainly in settings where the prevalence of both problems was already high before 2020.

In this scenario, population-based studies are important to better understand the association between FI and mental health amid Covid-19 pandemic, especially in low- and middle-income countries like Brazil, where social disparities were already large even before this public health issue. Therefore, we aimed to assess the association between household FI and MDE amid Covid-19 pandemic, using data from four consecutive population-based studies from a southern city of Brazil.

## Methods

### Setting, study’s sample and data collection

The data used in this study were obtained from a larger project aiming to investigate the prevalence of SARS-CoV-2 infection in the municipality of *Bagé*, *Rio Grande do Sul* state, Brazil. *Bagé* is a municipality located in the Brazilian farther south, along the border with Uruguay, and has around 120 000 inhabitants. In terms of socio-economic characteristics, *Bagé* presented lower *per capita* Gross Domestic Product (BRL 24 601·29 *v*. BRL 33 593·82 in the country), but a better development when compared with the country as a whole, with higher Human Development Index (0·740 *v*. 0·699; available at https://cidades.ibge.gov.br/brasil/rs/bage/panorama) and lower Gini coefficient (0·5708 *v*. 0·6086; available at http://tabnet.datasus.gov.br/cgi/ibge/censo/cnv/ginibr.def).

All individuals living in the urban area of *Bagé* were eligible to take part in this study. To select the sample, four consecutives and independent population-based surveys were conducted using a multi-stage sampling process in each of them. To ensure random and representative population sampling in the four population-based surveys, forty census tracts were randomly selected out of 126 existing in *Bagé*. In the second stage of the sampling process, ten households were selected in each census tract, totalling 400 visited households per survey. In the last stage, one individual per household was randomly selected to answer the questionnaire and take serological test for SARS-CoV-2, totalling 1600 eligible individuals to be interviewed in the four epidemiological surveys. It should be mentioned that 400 different households have been visited in each epidemiological survey wave, which assured four different samples in this study.

Data were collected in home interviews conducted by trained study personnel. Interviews and serological tests occurred between May (07–09 and 22–26 the first two surveys, respectively) and June 2020 (08–10 and 23–26 the last two surveys, respectively), led by trained community health agents and nursing technicians from the local public primary health care units. All the study personnel involved in data collection faced a training process for serological tests and questionnaire application with all of them wearing personal protective equipment during the fieldwork to avoid SARS-CoV-2 infection.

### Food insecurity status

In all epidemiological surveys, we assessed household FI status using a short-form version of the Brazilian Food Insecurity Scale proposed by Santos *et al.* ^(29)^. This short-form version is composed by five questions and, despite it does not allow to classify the different intensity levels of FI (mild, moderate and severe FI status), it allows screening of households experiencing this situation, presenting high sensitivity and specificity when compared with the complete questionnaire^(29)^. We applied this short-form version instead of the complete Food Insecurity Scale because we needed a quick questionnaire application in each household.

In our study, we made a small adaptation on the original questionnaire’s recall period: we used the beginning of the pandemic in Brazil (March, 2020) as the recall period of the FI questionnaire used in our study instead of the 3-month recall period proposed in the original version. We classified those households as food insecure whose selected interviewees reported at least one positive answer in the applied questions.

### Screening for major depressive episodes

We used the Patient Health Questionnaire-9 as screening for MDE. This questionnaire, which has been validated for the Brazilian Population^(30)^, assesses the frequency of MDE (depressive mood, anhedonia, trouble sleeping, tiredness or lack of energy, change of appetite or weight, feeling of guilt or uselessness, trouble concentrating, feeling slow or agitated and thoughts about death or suicidal ideation) in 2 weeks prior to the interview. The ten questions of the questionnaire are scored from 0 to 3 corresponding to ‘Never’, ‘Less than one week’, ‘One week or more’ and ‘Almost everyday’.

We used two approaches to define positive screening for MDE. Firstly, we used the cut-off point of ≥ 9 proposed by Santos *et al.* ^(30)^. This cut-off presented high sensitivity and specificity and has already been used in previous population-based studies in Brazil^(31,32)^. All those individuals who scored 9 or more in the test were then considered as having positive screening for MDE. Nevertheless, to increase the robustness of our study’s findings and considering the inherent limitations of the proposed cut-off point^(8,30)^, we also used the diagnostic criteria proposed by the Diagnostic and Statistical Manual of Mental Disorders (DSM-IV-TR), and classified a test as positive screening for MDE when five or more depressive symptoms have been present during the last 2 weeks, for at least 7 d with one of them being either depressive mood or anhedonia.

### Socio-demographic characteristics and measures of social distancing: potential confounders

Sex (male, female), age group (20–39, 40–59 and ≥ 60 years), educational level (none or elementary; high school; University education), skin colour (white, brown and black), household crowding (1 or 2 persons; 3 or 4 persons; 5 or more persons living in the visited household) and presence of younger than 18-year-old individuals in the household (yes or no) were the self-reported socio-demographic characteristics included in our analyses as co-variables.

We also included information on compliance to social distancing measures guided by municipality authorities and activities routine amid pandemic as co-variables of our study. Activities routine amid pandemic was collected through the question ‘How has your activities routine been during Covid-19 pandemic?’ (categorised as stayed at home; went out eventually; went out everyday). Compliance to social distancing measures guided by municipality authorities was evaluated using the question ‘How much do you think you are compliant with the social distancing measures guided by authorities?’ (categorised as very little or little; more or less; isolated).

### Statistical analysis

We used proportions and 95 % CI to describe sample’s characteristics as well as the prevalence of FI and MDE (overall and according to epidemiological survey wave). Statistically significant differences in MDE according to socio-demographic characteristics (sex, age, education, skin colour, household crowding and presence of younger than 18-year-old household members) and compliance to measures of social distancing (activities routine amid pandemic and social distancing) were assessed using chi-squared test of heterogeneity and non-parametric test for trends across ordered groups.

The association between FI status and MDE was based on models of crude and adjusted Poisson regression with robust variance, as analyses of cross-sectional studies with binary outcomes fit better using Poisson than logistic regression^(33)^. Adjusted Poisson regression models were assessed to check whether significant associations were independent of socio-demographic characteristics and compliance to measures of social distancing. We considered those co-variables as potential confounders associated with both MDE and FI at 20 % significance level (*P*-value ≤ 0·2). In all set of analyses, we used the goodness-of-fit test to evaluate if our tested Poisson models were appropriated. All analyses were performed using Stata16.1 and considered the complex study design.

## Results

From the 1600 individuals selected to take part in the study, we excluded three of them with incomplete questionnaires (0·2 % of overall sample). We also excluded forty-seven individuals younger than 20 years old (2·9 % of overall sample) because normally they are not responsible for household food purchases, which could interfere on FI questions. The final analyses included 1550 individuals who were interviewed in four consecutive epidemiological surveys (391, 390, 383 and 386 individuals in the first, second, third and fourth survey, respectively), and with available information on FI status and MDE. Women, elderly, those with low educational level (none or elementary school) and whites composed most of the sample. About 50 % reported to live in low crowded households (with, at most, two persons) and more than 35 % reported to live with younger than 18-year-old individuals at home. The socio-demographic characteristics of the sample were quite similar in all the four epidemiological surveys (see online supplementary material, Supplemental Table 1).

Regarding activities routine amid Covid-19 pandemic, less than one-fourth of the sample reported staying at home during social distancing guided by authorities. It is interesting to notice that between May and June 2020, the prevalence of individuals who reported staying at home decreased from 27·1 to 22·4 % (*P*-trend = 0·036). Additionally, more than 60 % stated to be social isolated, although this prevalence decreased from almost 70 % in the first wave to less than 55 % in the last wave of the study (*P*-trend < 0·001). Finally, 29·4 % of the sample presented household FI, with a slight decrease in this situation from wave 1 (35·2 %; 95 % CI 29·9 %, 41·0 %) to wave 4 (26·2 %; 95 % CI 19·5 %, 34·1) (see online supplementary material, Supplemental Table 1).

The prevalence of MDE varied according to the criteria used to define this condition in the Patient Health Questionnaire-9. When we used the cut-off point of ≥ 9, the overall prevalence of MDE was 9·6 % (95 % CI 7·3, 12·5), decreasing from the first (12·9 %; 95 % CI 9·6, 16·9) to the last wave of the survey (6·2 %; 95 % CI 4·0, 9·6). The prevalence of MDE was almost as twice as higher in women than men (11·5 % *v*. 6·1 %; *P*-value = 0·003), more frequent in younger individuals (20–39 years old) and in those living in overcrowded households (five or more residents). There were no significant associations between MDE and activities routine amid the pandemic or the compliance to social distancing measures (Table 1).

**Table 1:** Prevalence of major depressive episodes defined using the cut-off point of ≥ 9‡ according to socio-demographic characteristics, activities routine during the pandemic and compliance to social distancing measures guided by authorities, Bagé, Brazil, 2020 (*n* 1550)

Sample’s characteristics	Wave 1		Wave 2		Wave 3		Wave 4		Overall	
	%	95 % CI	%	95 % CI	%	95 % CI	%	95 % CI	%	95 % CI
Sex	0·229†		0·001†		0·113†		0·270†		0·003†	
Male	10·3	6·3, 16·5	2·9	1·1, 7·7	6·3	3·0, 12·7	4·2	1·7, 9·9	6·1	4·1, 8·9
Female	14·6	10·3, 20·5	11·6	6·9, 18·8	12·8	6·8, 22·8	6·8	4·3, 10·6	11·5	8·4, 15·4
Age (years)	0·955*		< 0·001*		0·879*		0·300*		0·033*	
20–39	13·8	7·8, 23·2	16·1	8·9, 27·3	11·2	4·9, 23·7	8·8	4·8, 15·7	12·4	8·9, 17·2
40–59	11·0	6·7, 17·6	8·6	3·3, 20·5	11·3	5·1, 23·0	5·6	2·8, 10·9	9·1	6·0, 13·5
≥ 60	13·9	8·5, 21·7	3·2	1·1, 8·7	10·7	5·6, 19·3	5·3	2·2, 12·3	8·2	5·5, 11·9
Education	0·294*		0·408*		0·575*		0·120*		0·227*	
Elementary	11·7	7·2, 18·0	9·5	4·7, 18·2	10·5	4·9, 21·2	4·1	1·9, 8·6	8·9	6·0, 13·1
High school	12·8	8·0, 19·8	6·1	3·2, 11·3	14·4	8·4, 23·6	8·6	4·7, 15·1	10·4	7·5, 14·2
Superior	17·5	9·6, 29·9	7·5	2·2, 22·3	11·1	3·7, 29·0	9·1	3·4, 22·2	11·7	7·2, 18·5
Skin colour	0·197†		0·177†		0·253†		0·764†		0·116†	
White	12·1	8·4, 17·0	7·2	3·9, 13·1	9·7	5·1, 17·6	6·1	3·7, 10·1	8·8	6·4, 11·9
Brown/Black	16·4	10·3, 25·3	12·4	6·2, 23·1	14·1	8·2, 23·2	5·1	1·5, 16·1	12·0	8·5, 16·6
Household crowding	0·085*		< 0·001*		0·347*		0·892*		0·017*	
1–2	8·2	5·1, 13·1	3·9	1·5, 9·4	12·6	7·2, 21·0	6·5	3·4, 12·0	7·9	5·4, 11·5
3–4	17·3	12·0, 24·3	8·3	4·5, 14·9	8·7	3·4, 20·7	6·3	3·1, 12·0	10·2	7·4, 13·8
≥ 5	13·0	5·4, 27·8	23·5	9·9, 46·4	9·8	3·5, 24·2	6·0	2·0, 16·9	13·3	8·4, 20·4
< 18-year-old household members	0·679†		0·004†		0·326†		0·382†		0·170†	
No	12·4	8·9, 16·9	4·5	2·1, 9·4	12·0	6·6, 20·7	5·3	2·8, 10·0	8·6	6·1, 12·0
Yes	13·8	8·6, 21·4	14·5	8·2, 24·3	9·0	4·7, 16·5	7·8	4·3, 13·6	11·3	8·1, 15·4
Activities routine amid pandemic	0·444†		0·889†		0·564†		0·662†		0·271†	
Stayed at home	16·2	10·2, 24·7	8·3	3·7, 17·7	13·6	6·5, 26·1	8·1	4·2, 15·1	11·8	8·2, 16·7
Went out eventually	12·1	7·7, 18·5	7·8	4·5, 13·2	11·2	5·4, 22·0	5·8	2·9, 11·3	9·2	6·6, 12·7
Went out everyday	10·8	6·2, 18·0	9·3	4·1, 20·0	8·3	4·4, 15·1	5·6	2·6, 11·3	8·3	5·5, 12·4
Social distancing	0·724†		0·290†		0·359†		0·171†		0·997†	
Very little/little	16·0	7·9, 29·7	4·6	1·2, 15·3	5·9	1·5, 20·3	10·1	5·2, 18·8	9·6	6·0, 15·1
More or less	11·4	6·1, 20·5	12·6	5·4, 26·8	8·6	4·7, 15·2	6·5	3·4, 12·3	9·5	6·4, 14·0
Isolated	12·9	9·3, 17·6	7·4	3·9, 13·7	12·6	6·5, 22·8	4·8	2·5, 9·1	9·7	7·1, 13·0
Total	12·9	9·6, 16·9	8·2	4·9, 13·5	11·0	6·4, 18·3	6·2	4·0, 9·6	9·6	7·3, 12·5

* *P*-values are displayed from a test from trend across ordered groups.

† *P*-values are displayed from chi-squared test.

‡ Positive screening for major depressive episodes defined using the cut-off point of ≥ 9 proposed by Santos *et al.* ^(30)^.

When we defined MDE using the DSM-IV-TR criteria, the overall prevalence dropped to 4·4 % (95 % CI 3·1, 6·0) (more than 50 % lower), with no differences among the survey’s wave. MDE prevalence was higher in women than men (5·2 % *v*. 2·8 %; *P*-value = 0·017). Twenty- to thirty-nine-year-old individuals, those with higher educational level and non-white skin colour ones (brown and black), also presented higher prevalence of MDE. Again, MDE defined using the DSM-IV-TR criteria were not associated with routine activities or social isolation during the Covid-19 pandemic (Table 2).

**Table 2:** Prevalence of major depressive episodes defined using the Diagnostic and Statistical Manual of Mental Disorders (DSM-IV-TR) criteria‡ according to socio-demographic characteristics, activities routine during the pandemic and compliance to social distancing measures guided by authorities, Bagé, Brazil, 2020 (*n* 1550)

Sample’s characteristics	Rodada 1		Rodada 2		Rodada 3		Rodada 4		Overall	
	%	95 % CI	%	95 % CI	%	95 % CI	%	95 % CI	%	95 % CI
Sex	0·333†		0·014†		0·237†		0·892†		0·017†	
Male	4·3	1·9, 9·2	0·7	0·1, 5·6	2·7	0·9, 8·2	3·6	1·3, 9·4	2·8	1·7, 4·6
Female	6·4	4·1, 10·0	5·4	2·9, 9·8	5·7	3·1, 10·2	3·3	1·6, 6·6	5·2	3·6, 7·4
Age (years)	0·137*		0·001*		0·961*		0·138*		0·002*	
20–39	7·2	3·5, 14·4	9·3	4·5, 18·4	3·4	1·1, 10·2	6·4	2·9, 13·6	6·6	4·1, 10·4
40–59	6·9	3·5, 13·2	3·6	1·0, 12·4	6·3	2·8, 13·7	2·9	1·1, 7·5	4·9	3·1, 7·8
≥ 60	3·0	1·1, 7·8	0·7	0·1, 4·7	4·0	1·7, 9·5	2·4	0·8, 7·2	2·5	1·4, 4·4
Education	0·195*		0·300*		0·927*		0·008*		0·012*	
Elementary	4·9	2·2, 10·4	3·2	1·0, 9·8	5·3	2·5, 10·8	1·4	0·4, 5·4	3·7	2·2, 6·1
High school	3·9	1·4, 10·4	3·4	1·4, 8·0	5·5	2·3, 12·6	4·7	2·2, 9·6	4·4	2·6, 7·3
Superior	10·9	5·1, 21·7	7·7	2·3, 22·6	5·6	1·5, 18·2	10·0	3·8, 23·9	8·9	5·2, 14·8
Skin colour	0·638†		0·217†		0·001†		0·794†		0·020†	
White	5·6	3·4, 8·9	3·0	1·6, 5·7	3·5	1·9, 6·3	3·3	1·7, 6·2	3·8	2·7, 5·4
Brown/Black	6·9	2·9, 15·7	6·2	2·6, 14·0	9·4	4·8, 17·7	2·7	0·7, 10·1	6·4	4·2, 9·7
Household crowding	0·839*		0·003*		0·213*		0·779*		0·321*	
1–2	3·4	1·5, 7·4	1·7	0·5, 5·2	6·1	3·1, 11·7	3·4	1·4, 8·2	3·7	2·3, 6·0
3–4	9·7	5·6, 16·3	3·3	1·4, 7·5	3·6	1·3, 9·8	3·6	1·4, 8·9	5·1	3·5, 7·3
≥ 5	0·0	0·0, 0·0	11·8	4·5, 27·5	2·4	0·3, 17·0	4·4	1·0, 17·0	4·7	2·1, 10·4
< 18-year-old household members	0·867†		0·003†		0·627†		0·481†		0·236†	
No	5·7	3·3, 9·6	1·2	0·4, 3·9	5·2	2·6, 10·2	3·0	1·3, 6·8	3·8	2·5, 5·8
Yes	5·3	2·6, 10·5	7·6	3·6, 15·3	3·8	1·3, 10·4	4·4	2·1, 9·3	5·3	3·4, 8·2
Activities routine amid pandemic	0·532†		0·529†		0·221†		0·920†		0·323†	
Stayed at home	6·9	3·5, 13·1	3·6	1·2, 10·4	7·4	3·1, 16·8	3·7	1·3, 10·0	5·5	3·5, 8·4
Went out eventually	5·9	3·2, 10·6	3·1	1·5, 6·2	4·9	2·6, 9·0	3·3	1·4, 7·6	4·2	3·0, 6·0
Went out everyday	3·4	1·1, 10·0	5·3	2·0, 13·7	2·1	0·5, 8·2	4·1	1·6, 10·4	3·7	2·1, 6·3
Social distancing	0·142†		0·777†		0·365†		0·079†		0·395†	
Very little/little	10·9	4·0, 26·1	2·3	0·4, 13·1	0·0	0·0, 0·0	8·2	3·6, 17·4	6,0	3·2, 10·9
More or less	1·5	0·2, 10·6	4·7	1·8, 11·6	4·3	1·7, 10·3	4·0	1·6, 9·7	3·7	2·2, 6·3
Isolated	5·8	3·5, 9·6	3·5	1·5, 8·3	5·5	3·0, 10·0	2·0	0·6, 6·4	4·4	3·1, 6·1
Total	5·6	3·6, 8·4	3·6	1·9, 6·8	4·7	2·7, 8·1	3·6	2·0, 6·4	4·4	3·1, 6·0

* *P*-values are displayed from a test from trend across ordered groups.

† *P*-values are displayed from chi-squared test.

‡ Positive screening for major depressive episodes defined using the DSM-IV-TR criteria, which classifies a test as positive screening when five or more depressive episodes are present for 1 week or more or everyday with one of them being either depressive mood or anhedonia.

Association between FI status and MDE evidenced higher prevalence of MDE in those individuals who experienced household FI. In the analysis using the cut-off point of ≥ 9, the prevalence of MDE was 15·8 % (95 % CI 12·4, 19·9) in those with FI and just 6·9 % (95 % CI 4·7, 10·2) in food secure individuals. When depressive episodes were assessed using the DSM-IV-TR criteria, the difference between groups was even larger: 8·2 % (95 % CI 5·7, 11·6) for those classified as food insecure and only 2·7 % (95 % CI 1·8, 4·2) for food secure ones (results not displayed in tables).

**Fig. 1:**
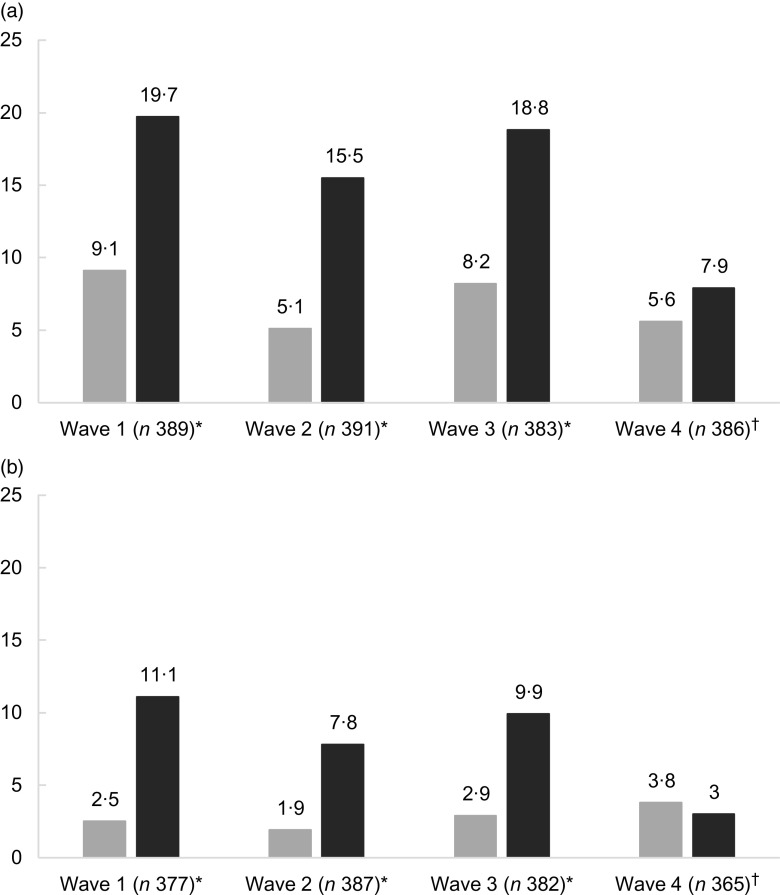
Prevalence of major depressive episodes using the cut-off point of ≥ 9 (a) and the Diagnostic and Statistical Manual of Mental Disorders criteria (b) according to household food insecurity/security status by epidemiological survey wave. *P-value from chi-squared test <0·05. ^†^ *P*-value from chi-squared test ≥0·05. 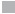, food secure; 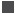, food insecure

Figure 1 shows the association between MDE and FI stratified by epidemiological survey wave. Independently of the way we used to define MDE, differences were only observed in the first three waves, which occurred, respectively, in the beginning and end of May, and in the beginning of June 2020: in both cases, MDE prevalence was almost 10 % points higher in the food insecure individuals. In the last survey, which occurred in the end of June, we observed a decrease in MDE in the food insecure individuals and significant differences between groups were not observed anymore (Fig. 1).

Table 3 shows results for crude and adjusted Poisson regression between FI and MDE, overall and stratified by epidemiological survey wave. Independent of the socio-demographic characteristics included as confounders in the tested models, MDE remained associated with FI status, being more than twice higher in those with FI, no matter the way we used to define the outcome. When we stratified by epidemiological survey wave, however, we observed that the association remained significant only in the first three waves when the outcome was defined using the cut-off point of ≥ 9, losing its significance in the last wave of the study. When MDE were defined using the DSM-IV-TR criteria, the adjusted associations were significant only in the first (beginning of May 2020) and third (beginning of June 2020) epidemiological survey waves (Table 3).

**Table 3:** Crude and adjusted Poisson regression models for the association between food insecurity and major depressive episodes, overall and according to epidemiological survey, Bagé, Brazil, 2020 (*n* 1550)

	Depressive symptoms diagnosed using the cut-off									
Food insecurity	Wave 1		Wave 2		Wave 3		Wave 4		Overall	
	PR	95 % CI	PR	95 % CI	PR	95 % CI	PR	95 % CI	PR	95 % CI
Crude analysis										
No	1·00		1·00		1·00		1·00		1·00	
Yes	2·2	1·3, 3·6	3·0	1·4, 6·6	2·3	1·2, 4·3	1·4	0·6, 3·2	2·3	1·6, 3·3
Adjusted analysis*										
No	1·0		1·0		1·0		1·0		1·00	
Yes	2·2	1·2, 3·9	2·3	1·2, 4·5	2·3	1·2, 4·5	1·5	0·6, 3·6	2·2	1·6, 3·2

	Depressive symptoms diagnosed using the DSM-IV-TR criteria									
Food insecurity	Wave 1		Wave 2		Wave 3		Wave 4		Overall	
	PR	95 % CI	PR	95 % CI	PR	95 % CI	PR	95 % CI	PR	95 % CI
Crude analysis										
No	1·00		1·00		1·00		1·00		1·00	
Yes	4·5	1·9, 10·6	4·2	1·8, 9·6	3·5	1·4, 8·6	0·8	0·2., 3·1	3·0	2·0, 4·5
Adjusted analysis†										
No	1·00		1·00		1·00		1·00		1·00	
Yes	5·1	1·8, 14·4	2·4	1·0, 5·7	2·9	1·1, 7·7	0·8	0·2, 3·9	2·6	1·8, 3·9

PR, prevalence ratio; DSM-IV-TR, Diagnostic and Statistical Manual of Mental Disorders.

* Adjusted for sex, age, skin colour, household crowding and the presence of younger than 18-year-old individuals.

† Adjusted for sex, age, level of education and skin colour.

## Discussion

Our study showed an association between FI status and MDE amid the Covid-19 pandemic using a population-based sample from a city located in southern Brazil. Using two different approaches to define MDE, we observed that FI increased the likelihood of depressive episodes, with those individuals who experienced this situation presenting a prevalence of MDE more than 2-fold higher than the food secure ones, independent of the socio-demographic characteristics included in the adjusted models.

Several investigations have demonstrated that FI is associated with different mental health problems, such as anxiety, stress and sleep disorders^(19,34–37)^. Nevertheless, according to a recently published meta-analysis, among the most common mental health disorders, depression is the one strongest associated with FI. Arenas *et al.* ^(38)^ have analysed data from forty-two studies that assessed cross-sectional association between FI and depression, and they have found a similar result to the one observed in our investigation: FI situation increased almost three times the odds of depression (OR = 2·74; 95 % CI 2·52, 2·97).

Despite association between FI and depression has been well described in the literature, the majority of studies about this relationship have been conducted with specific sub-groups such as low-income individuals, immigrants, people living with HIV, among others^(20,35,39)^. Results using samples from overall population, like our investigation, are scarcer but also indicate FI as an important determinant for the development of mental health disorders. For example, data from the National Longitudinal Study of Adolescent to Adult Health, conducted in the USA, evidenced that young adults who experienced FI presented greater odds of depression, anxiety and other mental health disorders^(40)^. Burrus *et al.* ^(41)^, using data from the National Health and Nutrition Examination Survey, have found that people living in FI situation were more likely to have major depressive disorder and, therefore, to attend mental health professionals with more frequency when compared with people without FI.

In Brazil, specifically, few population-based studies have investigated the association between FI and mental health disorders in overall population. Demenech *et al.* ^(42)^ found that FI status increased the prevalence of high perceived stress in 44 % in the city of *Rio Grande*, also located in Southern Brazil. In addition, Dumith *et al.* ^(43)^, using population-based data from the city of *Rio Grande* as well, revealed that FI status was associated with higher prevalence of suicidal thoughts (adjusted prevalence ratio = 1·75; 95 % CI 1·13, 2·72). It is important to highlight that both studies were carried out before the pandemic context and presented lower prevalence ratio when compared with the effect measures presented in our study. It might be suggesting that the effect of FI on mental health issues may be stronger amid Covid-19 pandemic, but more prospective population-based studies are necessary to confirm this assumption.

The mechanisms involved in the association between FI and depression are linked to several aspects, such as worrying and uncertainty of acquiring sufficient food supplies to maintain the energy necessities^(19,37)^ as well as shame of having no food at home^(44)^. In addition, severe FI and hunger may also lead to acquiring foods in socially unacceptable ways, which is associated with feelings of shame and guilty^(45)^, increasing the risk of anxiety and depression. Finally, FI is closely linked to poverty^(46)^, which is in turn associated with mental illness as well^(47)^. These mechanisms inside the pandemic frame, characterised by social isolation and fear of being infected by SARS-CoV-2, can further increase mental health symptoms and their consequences amid Covid-19 pandemic and afterwards.

Another interesting result of our study was the decrease in MDE prevalence in the fourth wave of the study, conducted in late June 2020, when MDE was defined using the cut-off of ≥ 9. Results stratified according to the FI status revealed that the decrease in MDE was based in the food insecure individuals. We have some hypothesis that may help to explain and understand this result. The first one is the payment of an emergency aid for families with economic struggling amid the pandemic, which started in March 2020 and helped more than 70 million people in Brazil throughout last year. We hypothesised that this emergency aid enabled food insecure individuals from our sample to buy staples and avoid hunger, diminishing the mental health symptoms associated with the lack of food in the last wave of our study. The National Household Sample Survey (PNAD Covid-19) conducted in 2020 has already demonstrated that the emergency aid decreased the extreme poverty in Brazil last year^(48)^. Nonetheless, the Brazilian government interrupted the emergency aid by the end of 2020 and FI situation in Brazil appears to have worsened after that. Results from a survey conducted by the Brazilian Research Network on Food Sovereignty and Security estimated that almost 55 % of the Brazilian population is experiencing FI situation in 2021, with 19 million Brazilians experiencing hunger (results available at https://pesquisassan.net.br/olheparaafome/).

A second hypothesis to explain the decrease in MDE among the food insecure participants is the return of labour activities after a large shutdown in the beginning of the pandemic. In Brazil, FI is more prevalent in younger, less educated and non-white individuals^(25,49)^, people who usually struggle to work from home. The return of labour activities might have helped individuals with FI to keep their jobs and salaries, avoiding the deterioration of mental health situation associated with unemployment and lack of food. In *Bagé*, specifically, a situation of public health emergency has been declared in March 2020, with suspension of education, social and cultural activities, although commercial establishments remained open to the population, according to the Municipality Decrees (available at http://www.bage.rs.gov.br).

Despite being associated with FI status, the overall prevalence of MDE in *Bagé* was not as different as the prevalence observed before the Covid-19 pandemic in Brazil. Results from the Global Burden of Disease Study 2017 pointed that the prevalence of depressive disorders, defined using the DSM-IV-TR, was 3·3 % (95 % CI 3·08 %, 3·57 %), with *Rio Grande do Sul* state presenting the second highest prevalence in the whole country (3·67 %)^(9)^. Munhoz *et al.* ^(8)^ using data from the 2013 Brazilian National Health Survey observed a similar prevalence of depressive episodes (4·1 %) using the same criteria proposed by the DSM-IV-TR to define this condition.

But different from our study, other published papers have reported an increase in mental health disorders during the pandemic in other countries^(15,16,50)^. In Brazil, more specifically in *Rio Grande do Sul* state, Feter *et al.* ^(14)^ have found an increase in symptoms of depression from 3·9 % before the Covid-19 pandemic to 29 % in June–July 2020. Notwithstanding being an important result, comparisons between this and our study should be made with cautions due to differences in the design and data collection between these two investigations. While Feter *et al.* ^(14)^ recruited their sample using an online-based platform, we interviewed our sample in face-to-face interviews. Additionally, young and highly educated individuals were overrepresented in their research, population sub-groups highly affected by unemployment and social isolation, both factors associated with higher risk of depression. Finally, they used a different tool to assess depression, and all these mentioned aspects may explain the differences in depression prevalence found in both studies.

Attention is needed to some limitations present in our study. The first one is the cross-sectional design employed here, precluding us to assess temporality in the analysed association. Longitudinal studies have shown that association between FI and mental health may operate in both directions^(28)^, increasing the risk of reverse causality in our paper. Furthermore, exposure and outcome were defined using screening tests, without possibility to diagnosis FI and depressive episodes. Nevertheless, the tools used in our study to define FI and MDE presented good results when compared with the gold standards. They are also easy and quickly to apply and they have been widely used in epidemiological studies^(35,49)^.

Finally, we need to discuss the implication in using the short-form version of the Brazilian Food Insecurity Scale instead of the complete questionnaire. Despite this 5-item tool being easy and quick to apply, which is imperative in an emergency scenario like Covid-19 pandemic, it does not detect the different intensity levels of FI, not allowing the authors to examine the prevalence of moderate and severe FI. It is a considerable limitation because we were not able to identify those individuals living in households with the worst scenario in food situation. Nonetheless, even without considering the different FI levels of intensity, this scale is important for tracking families living with this condition^(29)^, which may support calls for action to prevent and combat FI and hunger.

On the other hand, the use of two different ways to define MDE is a strength of our study, since both approaches presented similar results on the association with FI, increasing the robustness of our findings. Finally, to assess association between FI and MDE using population-based data from face-to-face interviews in the context of Covid-19 pandemic, where several studies have been conducted online, is another important positive point of our research.

In conclusion, FI status amid Covid-19 pandemic appears to be increasing the prevalence of MDE in a population-based sample from southern Brazil. The results presented in this research emphasise that actions need to be formulated to warrant basic living conditions to avoid FI and hunger and its consequences for the Brazilian population, especially in terms of mental health.

## Supplementary material

For supplementary material accompanying this paper visit https://doi.org/10.1017/S1368980021004626. click here to view supplementary material
