# Supplementary material for: Association between food insecurity and major depressive episodes amid Covid-19 pandemic: results of four consecutive epidemiological surveys from southern Brazil
Source: Public Health Nutr. 2021 Nov 24;25(4):944–53. doi: 10.1017/S1368980021004626 (PMC9991800; doi:10.1017/S1368980021004626)
Supplement: Supplementary file 1 [file S1368980021004626sup.zip › S1368980021004626sup002.docx]

**Supplementary information**

**Supplementary table 1.** Sociodemographic characteristics of the sample, activities routine during the pandemic and compliance to social distancing measures guided by authorities, overall and by epidemiological survey Bagé, Brazil, 2020 (N= 1550).

| **Sample’s characteristics** | **Wave 1**  **N (%)** | **Wave 2**  **N (%)** | **Wave 3**  **N (%)** | **Wave 4**  **N (%)** | **Overall sample** |
| --- | --- | --- | --- | --- | --- |
| Sex |  |  |  |  |  |
| Male | 145 (37.8) | 136 (36.0) | 111 (29.5) | 120 (32.5) | 512 (34.0) |
| Female | 239 (62.2) | 242 (64.0) | 265 (70.5) | 249 (67.5) | 995 (66.0) |
|  |  |  |  |  |  |
| Age (years) |  |  |  |  |  |
| 20-39 | 116 (29.8) | 87 (22.7) | 89 (22.4) | 102 (27.1) | 394 (25.8) |
| 40-59 | 136 (35.0) | 139 (36.3) | 142 (37.3) | 143 (38.0) | 560 (36.6) |
| ≥60 | 137 (35.2) | 157 (41.0) | 150 (39.4) | 131 (34.8) | 575 (37.6) |
|  |  |  |  |  |  |
| Educational level |  |  |  |  |  |
| Elementary | 147 (43.6) | 158 (45.7) | 152 (45.5) | 147 (43.0) | 604 (44.4) |
| High school | 133 (39.5) | 148 (42.8) | 146 (43.7) | 140 (40.9) | 567 (41.7) |
| Superior | 57 (16.9) | 40 (11.6) | 36 (10.8) | 55 (16.1) | 188 (13.8) |
|  |  |  |  |  |  |
| Skin colour |  |  |  |  |  |
| White | 298 (80.3) | 304(80.0) | 290 (77.3) | 293 (78.8) | 1185 (78.8) |
| Brown/Black | 73 (19.7) | 81 (20.0) | 85 (22.7) | 79 (21.2) | 179 (11.9) |
|  |  |  |  |  |  |
| Household crowding |  |  |  |  |  |
| 1-2 persons | 182 (47.2) | 181 (46.5) | 199 (57.7) | 185 (48.8) | 747 (48.8) |
| 3-4 persons | 150 (38.9) | 157 (40.4) | 138 (36.5) | 144 (38.0) | 589 (38.4) |
| 5 or more persons | 54 (14.0) | 51 (13.1) | 41 (10.9) | 50 (13.2) | 196 (12.8) |
|  |  |  |  |  |  |
| <18-year-old household members |  |  |  |  |  |
| No | 252 (64.5) | 246 (62.9) | 250 (65.3) | 244 (63.2) | 992 (64.0) |
| Yes | 138 (35.5) | 145 (37.1) | 133 (34.7) | 142 (36.8) | 558 (36.0) |
|  |  |  |  |  |  |
| Activities routine amid pandemic |  |  |  |  |  |
| Stayed at home | 105 (27.1) | 84 (21.6) | 81 (21.2) | 86 (22.4) | 356 (23.1) |
| Went out eventually | 190 (49.0) | 230 (59.1) | 205 (53.7) | 190 (49.5) | 815 (52.8) |
| Went out everyday | 93 (24.0) | 75 (19.3) | 96 (25.1) | 108 (28.1) | 372 (24.1) |
|  |  |  |  |  |  |
| Social distancing |  |  |  |  |  |
| Very little/little | 50 (13.0) | 44 (11.3) | 34 (8.9) | 69 (18.0) | 197 (12.8) |
| More or less | 70 (18.2) | 87 (22.4) | 93 (24.4) | 107 (27.9) | 357 (23.2) |
| Isolated | 264 (68.8) | 257 (66.2) | 255 (66.8) | 207 (54.1) | 983 (64.0) |
|  |  |  |  |  |  |
| Household food insecurity |  |  |  |  |  |
| Yes | 137 (35.2) | 116 (29.7) | 101 (26.4) | 101 (26.2) | 455 (29.4) |
| No | 253 (64.8) | 275 (70.3) | 282 (73.6) | 285 (73.8) | 1095 (70.7) |
|  |  |  |  |  |  |
| **Total** | **390 (100.0)** | **391 (100.0)** | **383 (100.0)** | **386 (100.0)** | **1550 (100.0)** |

Maximum percentage of unknown observation: 191(12.3%) for educational level.
